# A corpus-based investigation of the English translations of Mao Zedong’s speeches

**DOI:** 10.3389/fpsyg.2022.1071064

**Published:** 2022-11-24

**Authors:** Libo Huang, Xinyu Shi

**Affiliations:** Foreign Language and Literature Institute, Xi’an International Studies University, Xi’an, China

**Keywords:** English translations of Mao Zedong’s speeches, normalized spoken texts, original spoken texts, linguistic features, directionality of translation

## Abstract

This study adopts a corpus-based approach to examine the linguistic features manifested in the English translations of Mao Zedong’s speeches, taking Winston Churchill’s speeches (representative of normalized spoken texts) and the spoken texts in BNC Sampler (representative of original spoken texts) as the reference corpora. By investigating the macro- and micro-linguistic features, it is found that the translated Mao’s speeches (both direct and inverse translations) differ from normalized spoken texts as well as original spoken texts in three aspects: (i) macro-linguistic features, (ii) the use of personal pronouns, (iii) the use of modal verbs. In terms of macro-linguistic features, the average word length of the English translations is higher than that of normalized spoken texts and that of original spoken texts; the standardized type/token ratio and average sentence length of the English translations are higher than those of original spoken texts, but lower than those of normalized spoken texts. Meanwhile, in terms of the use of personal pronouns, the English translations of Mao’s speeches prefer the underuse of the first person singular pronoun *I*. Furthermore, as far as modal verbs are concerned, the English translations of Mao’s speeches prefer the overuse of *must* and *should* on the one hand, and the underuse of *shall*, *could*, *may*, and *would* on the other hand. Therefore, it can be said that the translated Mao’s speeches exhibit some particular linguistic features, which can not only be differentiated from normalized spoken texts, but also be distinguished from original spoken texts. They are in a middle position in relation to normalized spoken texts as well as original spoken texts. This in-betweenness not only exhibits Mao’s creative and idiosyncratic language use, but also reflects the influence of the language transfer from Chinese into English.

## Introduction

A speech text can be the draft for an oral delivery, or the record of an unprepared oral presentation. Linguistically speaking, speech texts can be categorized as spoken or written languages. Yet such a categorization seems too general and does cause some problems in investigating this type of text. As a matter of fact, speech texts exhibit the characteristics of both spoken and written languages, which has been under-explored so far. Speeches or statements made by political leaders or delivered on political occasions are usually designated as “political writings,” “political texts,” or “political discourse,” all of which are vague, umbrella terms and cover a variety of text types or genres (Trosborg, 1997: 145; Schäffner, 1997a,b: 119–120). In some studies, speech texts are often equated with political discourse and are discussed from different perspectives in the field of translation studies (Baumgarten and Gagnon, 2005; Schäffner and Bassnett, 2010; Schäffner, 2012; Bánhegyi, 2014; Wang and Feng, 2018; Du and Chen, 2022, etc.). Nevertheless, a more explicit linguistic delimitation of speech texts is needed, based on the medium of communication and the form of presentation.

Quite a few texts in Mao Zedong’s writings are based on his oral speeches. Many texts, originally delivered without prepared scripts, have evolved from speeches into textual classics, including “On the 10 Major Relationships” “On the Correct Handling of Contradictions Among the People” “Talk at an Enlarged Working Conference” “Rectify the Party’s Style of Work” “Oppose the Stereotyped Party Writing” “Serve the People” “In Memory of Norman Bethune” “The Foolish Old Man Who Removes the Mountains” (Chen, 2020: 227). The evolutionary process from oral delivery to written classics indicates that Mao’s speech texts we read today are a fusion of both spoken and written language. In terms of content, these texts often differ from the original oral delivery in diction; stylistically, these texts can reflect not only the style of written language but also that of spoken language. In this sense, Mao’s speech texts cannot be simply categorized as purely written or spoken texts. Similarly, the translations of Mao’s speech texts also cannot be classified into purely written or spoken texts.

Focusing on the English translations of Mao’s speech texts, this study examines the differences between the direct and inverse translations in terms of macro-linguistic features, personal pronouns and modal verbs. Here, direct translation, according to Shuttleworth and Cowie (1997: 41), is “the type of translation in which the translator works into, rather than away from, his or her native language (or language of habitual use),” while “the opposite procedure is termed inverse translation or service translation.” As far as the English translations of Mao’s speeches are concerned, both direct and inverse translations are involved in the present study. Two corpora of spoken English texts, namely Winston Churchill’s speeches and the spoken texts in BNC Sampler, are employed as reference corpora to decide the distinctiveness of the English translations of Mao’s speeches. The comparisons are mainly carried out intra-lingually: (i) between the direct and inverse translations, (ii) between the three English translations and Churchill’s speeches, and (iii) between the three English translations and the spoken texts in BNC Sampler.

## Speaking vs. writing, spoken forms vs. written forms

Speaking and writing are the two necessary means of communication, thus resulting in the two varieties of language: spoken and written languages. Spoken language comes from the production of speech, while written language is made up of written symbols such as letters, numbers and punctuation marks, etc. Many studies have discussed the differences between those two language varieties (Halliday, 1989; Cornbleet and Carter, 2001; Biber et al., 2002; Aijmer and Stenström, 2004; Biber and Barbieri, 2007; Biber, 2009; Gray and Biber, 2013). For example, in style, spoken language is usually more casual, with the common use of colloquial expressions. But written language tends to be more formal, involving the carefully chosen lexis and grammar. In addition, spoken language is in most cases produced on the spot, but written language can be edited for many times before its final version.

Based on the medium of communication, language can be presented in two basic forms: spoken and written form. In a broad sense, spoken form generally corresponds to spoken language, and written form to written language. However, it should be noted that this general correspondence does not include all the specific cases.

The fact is that presentation forms (spoken and written forms) do not completely equate to language varieties (spoken and written language). As is pointed out by Ye (1990: 134), “written language is not equivalent to written forms. When spoken language is transcribed into writings, the writings become the written forms of spoken language; similarly, written language also has its spoken forms.” In other words, presentation forms (spoken and written forms) may intermingle with language varieties (spoken and written language), resulting in four types including (i) spoken language in spoken forms, (ii) written language in written forms, (iii) spoken language in written forms, and (iv) written language in spoken forms. Stylistically speaking, the last two types are hybrid languages, since they are the mixtures of written and spoken language.

“Writing and speaking are not at opposite ends of the spectrum” (Cornbleet and Carter, 2001: 92). Therefore, it is not surprising that the hybrid languages contain the features of both written and spoken language. Originating from speech, spoken language in written forms, more often than not, is unable to remove the innate linguistic features of spoken language. On the other hand, presented in the texts, spoken language in written forms makes use of the written symbols (rather than sounds) to express particular meanings. In this sense, spoken language in written forms has to bear the features of both written and spoken language.

## Original spoken texts and normalized spoken texts

Spoken language in written forms, one type of the hybrid languages, can be further divided into two subtypes, according to the stylistic features the texts exhibit. The first subtype, referred to as “original spoken texts” in the present study, is the text that accurately records the exact language use of an oral presentation, thus reflecting only the features of the spoken language. That is to say, the content of the text remains identical to that of the actual oral delivery. Take the spoken texts in the British National Corpus (BNC) for instance. Those texts, containing pauses, silences, fillers, grammatical errors, etc., are

Yes. We shall just have to pretend it’s not there, there’s not, there’s not quite as bad as when I had to speak for Amnesty on Radio Essex last year and it was live, as every word, every word I spoke was being you know being heard by a lot of people and that’s, that was very, that was very intimidating.

(Extracted from the BNC Sampler, XML version, 2005: text identifier DCH, sentence No. 1-2)

accurate and detailed transcriptions of oral delivery in a variety of communicative contexts. Human interventions in the authenticity of the texts are limited as much as possible.

The other subtype of spoken language in written forms, referred to as “normalized spoken texts” in the present study, is the text which adapts the language use of an oral presentation to written forms. Such texts combine the features of both spoken and written languages, and thus differ somewhat from the actual oral delivery owing to human interventions. For example, the texts contained in the book *Never Give In! The Best of Winston Churchill’s Speeches* (Churchill, 2004) are revised on the basis of Winston Churchill’s speech content. Those texts exclude pauses, silences, fillers or

Though Parliament is dull, it is by no means idle. A measure is before them of the greatest importance to the working men of this country. I venture to hope that, if you think it presumptuous in one so young to speak on such a subject, you will put it down to the headstrong enthusiasm of youth.

(Churchill, 2004: 3)

grammatical errors in spoken language, and thus are not completely consistent with the original delivery. Another example is Mao Zedong’s “Talks at the Yenan Forum on Literature and Art,” which has developed many textual forms including a speech outline, minutes, a proofread script, a published draft with revisions, and revised editions (Qiu, 2020: 99). It originally developed from a speech outline prepared for the Yan’an Forum on Literature and Art in May 1942 (Hu, 2014: 263; Zhou, 2022: 21–22). On May 2 and May 23, Mao made two speeches which were recorded in minutes. After the forum, the minutes of Mao’s speech were proofread by Qiaomu Hu (Mao’s secretary), producing an early version of the spoken text (Zhou, 2022: 22). On 19 October 1943, *Jiefang Ribao* (Liberation Daily, a newspaper run by the Communist Party of China at Ya’an) published this text on the first, second and fourth pages, which was the first open publication of the text (Jin, 2005: 77). Almost 10 years later, in 1953, this text was further revised and then included in the third volume of *Mao Zedong Xuanji* (Pan, 1982: 1). Therefore, the above-listed textual forms all develop from Mao’s speech into different versions. And these written texts, some of which are revised many times after the oral delivery, embody the features of both spoken and written languages to varying degrees.

In this sense, Mao’s speech texts are normalized spoken texts, and their translations are thus translated normalized spoken texts. It is, therefore, an interesting question as to whether the translated normalized spoken texts contain some peculiar linguistic features. This study adopts a corpus-based approach to investigate the linguistic features of the English translations of Mao’s speeches, taking Winston Churchill’s speech texts (representative of normalized spoken English texts) and the spoken texts in BNC Sampler (representative of original spoken English texts) as the reference corpora. The linguistic features will be examined at two levels: at the macro-linguistic level, general statistical data (including standardized type/token ration, average word length and average sentence length) will be analyzed; at the micro-linguistic level, the use of function words (including personal pronouns and modal verbs) will be investigated.

The research questions are formulated as follows:

1. At the macro-linguistic level, what features do the English translations of Mao’s speeches present compared with Churchill’s speeches and the spoken texts in BNC Sampler? What are the probable causes for the differences?
2. At the micro-linguistic level, i.e., the use of personal pronouns and modal verbs, to what extent do the English translations of Mao’s speeches differ from Churchill’s speech and the spoken texts in BNC Sampler, respectively? What are the probable causes for the differences?
3. As far as the English translations are concerned, in which aspects do the translations of Mao’s speech texts in SWM (inverse translation) differ from those in CWM and MRP (direct translations)? And can the English translations of Mao’s speeches be differentiated from Churchill’s speech and the spoken texts in BNC Sampler?

## Data and methodology

Among various English translations of Mao Zedong’s writings, the most representative ones include: (i) *Selected Works of Mao Tse-tung* (Mao, 1961, 1965a,b,c), hereinafter referred to as SWM, (ii) *Collected Works of Mao Tse-tung* (*1917–1919*) (Mao, 1978), hereinafter referred to as CWM, (iii) *Mao’s Road to Power: Revolutionary Writings 1912–1949* (Schram, 1992; Schram and Hodes, 1994, 1995, 1997, 1999, 2004, 2005; Schram et al., 2015), hereinafter referred to as MRP. SWM is initiated by the Chinese government and published by Foreign Languages Press in Beijing in the 1960s; CWM is completed and published by the U.S. Joint Publications Research Service (JPRS) in 10 volumes in 1978; MRP is funded by the National Endowment for the Humanities in the U.S. and edited by Stuart Reynolds Schram. That is to say, the project of SWM is organized by the Chinese government, while CWM and MRP are translation programs in the U.S. at different historical periods. Thus, in terms of the directionality of translation, SWM belongs to the category of inverse translation (that is, translation into a foreign language), while CWM and MRP are direct translations (that is, translation into the translators’ mother tongue).

In this study, the translated Mao’s speeches are extracted from the three English translations (SWM, CWM and MRP), so as to examine the discrepancies in linguistic characteristics between direct and inverse translations. Both macro- and micro-linguistic features are taken into account. Besides, Churchill’s speech texts (hereinafter referred to as WCH) and the spoken texts in BNC Sampler (hereinafter referred to as BNC-S) are used as reference corpora. WCH is representative of the normalized spoken English texts produced by native English-speaking state leaders, while BNC-S represents the original spoken English texts. The purpose of comparing the English translations of Mao’s speeches with WCH and BNC-S is to examine the similarities and differences between translated normalized spoken texts, normalized spoken English texts and original spoken English texts.

There are 35 translated normalized spoken texts (about 210,000 words) in SWM, 26 (about 100,000 words) in CWM and 71 (about 380,000words) in MRP. It was found that some of the translations in MRP are mainly based on those corresponding texts in SWM, causing overlap between MRP and SWM. To ensure the comparability between those corpora, we left out those overlapping texts in MRP, and excluded some excessively long texts from SWM. The finalized texts under investigation are: 30 translated normalized spoken texts in SWM, 26 in CWM and 44 in MRP (see Supplementary Appendices).

With the assistance of WordSmith Tools Version 6.0 (Scott, 2015), this study investigates the macro- and micro-linguistic features of the three English translations, taking WCH and BNC-S as the reference corpora. Firstly, it makes a comparative analysis of the macro-linguistic statistics of the three English translations. And then, the study explores the use of personal pronouns and modal verbs in the three English translations.

## Data analysis

### Macro-linguistic features

Table 1 summarizes the macro-linguistic statistics of the English translations of Mao’s speeches, WCH, and BNC-S. There are some differences between the different types of spoken texts in macro-linguistic statistics, including standardized type/token ratio (STTR), average word length (AWL), average sentence length (ASL).

**Table 1:** Macro-linguistic statistics of different types of spoken texts.

Texts	Tokens	Types	TTR	STTR	AWL	Num. of sentences	ASL
SWM	96,710	6,225	6.44	37.16	4.80	4,265	22.68
CWM	96,233	5,781	6.01	35.90	4.97	4,206	22.88
MRP	73,676	5,468	7.42	35.93	4.71	3,924	18.78
WCH	186,003	11,149	5.99	43.22	4.52	7,196	25.85
BNC-S	947,843	20,968	2.21	33.17	4.00	77,375	12.25

In terms of STTR, CWM (35.90) is very close to MRP (35.93), while SWM (37.16) is higher than CWM and MRP. This indicates that the direct translations CWM and MRP are similar in lexical richness and readability, and the inverse translation SWM is featured by a higher degree of lexical richness and yet a lower degree of readability. Compared with the reference corpora, the STTRs of the three English translations are lower than that of WCH (43.22) but higher than that of BNC-S (33.17). This shows that, on the one hand, the three English translations demonstrate relatively less lexical richness but more readability than normalized spoken English texts; on the other hand, they show more lexical richness but less readability than original spoken English texts.

Regarding of AWL, SWM (4.80) is lower than CWM (4.97) and higher than MRP (4.71), and all the three English translations are higher than WCH (4.52) and BNC-S (4.00). It can be found that the spoken texts in inverse and direct translations are relatively similar in terms of AWL. Meanwhile, the three English translations are close to normalized spoken English texts in AWL, differentiating themselves from original spoken English texts.

In terms of ASL, SWM (22.68) is lower than CWM (22.88) but higher than MRP (18.78), with SWM and CWM being very close to each other. The ASLs of all the three English translations are lower than that of WCH (25.85) but much higher than that of BNC-S (12.25). This shows that in terms of ASL, the three English translations of Mao’s speeches reflect a more formal and serious style of normalized spoken English texts, which is different from that of original spoken English texts.

### The use of personal pronouns

Based on the statistics provided by WordSmith Tools Version 6.0 (Scott, 2015), this section investigates the three English translations in the use of personal pronouns. It firstly conducts a comparison among the three English translations. On that basis, in order to explore their relation to normalized spoken English texts and original spoken English texts, the three English translations are further compared with WCH and BNC-S.

Tables 2, 3 list the key personal pronouns in SWM, in comparison with CWM and MRP, respectively. There are significant differences in the use of personal pronouns between inverse and direct translations. The pronouns such as *we*, *you*, *he*, and *I* are all significantly overused in SWM compared with CWM, while all the four personal pronouns are significantly underused in SWM compared with MRP, although the three English translations of Mao’s speeches are consistent in style, register and text type.

**Table 2:** Key personal pronouns in SWM (in comparison with CWM).

No.	Personal pronouns	SWM		CWM		Keyness	Significance
		Freq.	%	Freq.	%		
3	We	1,173	1.21	722	0.74	107.62	0.00
5	You	139	0.14	21	0.02	85.55	0.00
64	He	141	0.15	68	0.07	24.78	0.00
98	I	151	0.16	86	0.09	17.26	0.00

**Table 3:** Key personal pronouns in SWM (in comparison with MRP).

No.	Personal pronouns	SWM		MRP		Keyness	Significance
		Freq.	%	Freq.	%		
203	It	828	0.85	725	0.98	−7.37	0.01
296	We	1,173	1.21	1,111	1.50	−27.17	0.00
310	He	141	0.15	206	0.28	−36.12	0.00
315	I	151	0.16	226	0.31	−42.22	0.00
321	You	139	0.14	353	0.48	−162.05	0.00

Concerning the use of personal pronouns, then, which of the three translations is close to normalized spoken English texts and at the same time distinct from and original spoken English texts? It is therefore necessary to compare the three English translations with WCH and BNC-S, respectively.

Tables 4–6 list the key personal pronouns in SWM, CWM and MRP, in comparison with WCH. Compared with normalized spoken English texts, which are represented by WCH, the three English translations exhibit the following characteristics in the use of personal pronouns. Firstly, the first person plural pronoun *we* is significantly underused in the CWM while significantly overused in the MRP. There is no significant difference in the frequency of *we* between the SWM and the WCH. Secondly, the second person pronoun *you* is significantly underused in both SWM and CWM, while it is significantly overused in MRP. Thirdly, the first person singular pronoun *I* is significantly underused in all the three English translations.

**Table 4:** Key personal pronouns in SWM (in comparison with WCH).

No.	Personal pronouns	SWM		WCH		Keyness	Significance
		Freq.	%	Freq.	%		
831	She	8	—	55	0.03	−12.03	0.00
868	It	828	0.85	1,925	1.03	−21.00	0.00
906	You	139	0.14	487	0.26	−39.69	0.00
912	He	141	0.15	502	0.27	−42.70	0.00
948	I	151	0.16	1,886	1.01	−653.74	0.00

**Table 5:** Key personal pronouns in CWM (in comparison with WCH).

No.	Personal pronouns	CWM		WCH		Keyness	Significance
		Freq.	%	Freq.	%		
890	It	865	0.89	1,925	1.03	−12.68	0.00
1,046	He	68	0.07	502	0.27	−124.92	0.00
1,053	We	722	0.74	2,413	1.29	−175.35	0.00
1,054	You	21	0.02	487	0.26	−203.13	0.00
1,060	I	86	0.09	1,886	1.01	−784.50	0.00

**Table 6:** Key personal pronouns in MRP (in comparison with WCH).

No.	Personal pronouns	MRP		WCH		Keyness	Significance
		Freq.	%	Freq.	%		
70	You	353	0.48	487	0.26	76.35	0.00
327	We	1,111	1.50	2,413	1.29	17.11	0.00
825	I	226	0.31	1,886	1.01	−326.91	0.00

Tables 7–9, respectively, list the key personal pronouns in SWM, CWM and MRP, in comparison with BNC-S. In terms of the use of personal pronouns, the three English translations share some similarities and differences with original spoken English texts, represented by BNC-S. First, the first person plural pronoun *we* is significantly underused in CWM. Second, the four personal pronouns *I, you, it*, and *he* are significantly underused in all the three English translations.

**Table 7:** Key personal pronouns in SWM (in comparison with BNC-S).

No.	Personal pronouns	SWM		BNC-S		Keyness	Significance
		Freq.	%	Freq.	%		
247	We	1,173	1.21	7,846	0.83	147.88	0.00
1942	He	141	0.15	5,350	0.56	−295.66	0.00
1945	She	8	—	3,417	0.36	−333.70	0.00
1952	It	828	0.85	18,229	1.92	−564.05	0.00
1953	You	139	0.14	22,311	2.35	−2048.01	0.00
1954	I	151	0.16	24,944	2.63	−2304.60	0.00

**Table 8:** Key personal pronouns in CWM (in comparison with BNC-S).

No.	Personal pronouns	CWM		BNC-S		Keyness	Significance
		Freq.	%	Freq.	%		
1763	We	722	0.74	7,846	0.83	−7.58	0.01
1959	He	68	0.07	5,350	0.56	−416.44	0.00
1961	It	865	0.89	18,229	1.92	−522.71	0.00
1965	You	21	0.02	22,311	2.35	−2289.53	0.00
1966	I	86	0.09	24,944	2.63	−2435.30	0.00

**Table 9:** Key personal pronouns in MRP (in comparison with BNC-S).

No.	Personal pronouns	MRP		BNC-S		Keyness	Significance
		Freq.	%	Freq.	%		
87	We	1,111	1.50	7,846	0.83	356.63	0.00
1,372	He	206	0.28	5,350	0.56	−103.59	0.00
1,397	It	725	0.98	18,229	1.92	−335.97	0.00
1,401	You	353	0.48	22,311	2.35	−1115.40	0.00
1,402	I	226	0.31	24,944	2.63	−1546.71	0.00

### The use of modal verbs

Based on the lexical usage statistics retrieved from WordSmith Tools Version 6.0 (Scott, 2015), this section compares the three English translations in the use of modal verbs. The comparison is preliminarily conducted among the three English translations. On that basis, in order to explore their relation to normalized spoken English texts and original spoken English texts, the three English translations are further compared with WCH and BNC-S.

Tables 10, 11 list the key modal verbs in SWM (in comparison with CWM and MRP respectively). As can be seen from the two tables, the use of modal verbs in the inverse translation SWM shows the following features: (i) *shall* and *can* are significantly overused in SWM, compared with CWM and MRP; (ii) *could* is significantly overused in SWM compared with CWM, while it is significantly underused in SWM compared with MRP; (iii) *must* is significantly overused in SWM compared with MRP; (iv) *would*, *may* and *ought* are significantly underused in SWM compared with CWM. On the whole, the differences between the inverse translation SWM and the direct translation CWM are more obvious.

**Table 10:** Key modal verbs in SWM (in comparison with CWM).

No.	Modal verbs	SWM		CWM		Keyness	Significance
		Freq.	%	Freq.	%		
36	Shall	89	0.09	25	0.03	34.80	0.00
47	Can	317	0.33	190	0.20	31.31	0.00
50	Would	103	0.11	37	0.04	30.16	0.00
56	May	105	0.11	41	0.04	27.16	0.00
157	Ought	14	0.01	0	−	12.06	0.00
187	Should	381	0.39	296	0.31	10.40	0.00
240	Could	32	0.03	12	0.01	8.19	0.00

**Table 11:** Key modal verbs in SWM (in comparison with MRP).

No.	Modal verbs	SWM		MRP		Keyness	Significance
		Freq.	%	Freq.	%		
7	Must	536	0.55	237	0.32	49.66	0.00
37	Shall	89	0.09	25	0.03	20.27	0.00
147	Can	317	0.33	186	0.25	7.84	0.01
201	Could	32	0.03	46	0.06	−7.23	0.01

Tables 12–14, respectively, list the key modal verbs in SWM, CWM and MRP, in comparison with WCH. It can be found that, compared with normalized spoken English texts, *should* and *must* are significantly overused, while *shall*, *could*, *may*, and *would* are significantly underused in all the three English translations. At the same time, *might* and *ought* are significantly underused in SWM and MRP, and *can* is significantly overused in SWM.

**Table 12:** Key modal verbs in SWM (in comparison with WCH).

No.	Modal verbs	SWM		WCH		Keyness	Significance
		Freq.	%	Freq.	%		
21	Must	536	0.55	408	0.22	213.18	0.00
91	Should	381	0.39	417	0.22	64.44	0.00
178	Can	317	0.33	394	0.21	33.57	0.00
787	Shall	89	0.09	246	0.13	−8.39	0.00
796	Ought	14	0.01	65	0.03	−8.83	0.00
914	Could	32	0.03	206	0.11	−44.74	0.00
918	Might	8	−	130	0.07	−48.29	0.00
926	May	105	0.11	464	0.25	−62.25	0.00
937	Would	103	0.11	553	0.30	−99.34	0.00

**Table 13:** Key modal verbs in CWM (in comparison with WCH).

No.	Modal verbs	CWM		WCH		Keyness	Significance
		Freq.	%	Freq.	%		
29	Must	537	0.55	408	0.22	214.60	0.00
438	Should	296	0.30	417	0.22	16.65	0.00
1,026	Shall	25	0.03	246	0.13	−74.10	0.00
1,029	Could	12	0.01	206	0.11	−78.57	0.00
1,050	May	41	0.04	464	0.25	−151.80	0.00
1,055	Would	37	0.04	553	0.30	−203.73	0.00

**Table 14:** Key modal verbs in MRP (in comparison with WCH).

No.	Modal verbs	MRP		WCH		Keyness	Significance
		Freq.	%	Freq.	%		
194	Should	254	0.34	417	0.22	29.16	0.00
264	Must	237	0.32	408	0.22	21.77	0.00
735	Could	46	0.06	206	0.11	−12.26	0.00
739	Ought	6	−	65	0.03	−12.93	0.00
800	Might	4	−	130	0.07	−41.33	0.00
804	Shall	25	0.03	246	0.13	−48.08	0.00
816	Would	76	0.10	553	0.30	−81.69	0.00
818	May	52	0.07	464	0.25	−84.40	0.00

Tables 15–17, respectively, list the key modal verbs in SWM, CWM and MRP, in comparison with BNC-S. It can be observed that the three English translations show the following characteristics in terms of the use of modal verbs: (i) *must*, *should*, *shall*, and *may* are significantly overused in SWM, while *must* and *should* are significantly overused in CWM and MRP; (ii) both *could* and *would* are underused in the three English translations; (iii) *might* is underused in SWM, *can* is underused in CWM, and *can* and *might* are underused in MRP.

**Table 15:** Key modal verbs in SWM (in comparison with BNC-S).

No.	Modal verbs	SWM		BNC-S		Keyness	Significance
		Freq.	%	Freq.	%		
14	Must	536	0.55	616	0.06	1,891.05	0.00
74	Should	381	0.39	1,029	0.11	523.80	0.00
386	Shall	89	0.09	291	0.03	88.24	0.00
669	May	105	0.11	500	0.05	45.65	0.00
1874	Might	8	−	691	0.07	−54.17	0.00
1904	Would	103	0.11	2,524	0.27	−89.65	0.00
1906	Could	32	0.03	1,538	0.16	−97.40	0.00

**Table 16:** Key modal verbs in CWM (in comparison with BNC-S).

No.	Modal verbs	CWM		BNC-S		Keyness	Significance
		Freq.	%	Freq.	%		
14	Must	537	0.55	616	0.06	1,898.98	0.00
178	Should	296	0.30	1,029	0.11	266.39	0.00
1896	Can	190	0.20	3,535	0.37	−77.43	0.00
1917	Could	12	0.01	1,538	0.16	−132.62	0.00
1932	Would	37	0.04	2,524	0.27	−186.65	0.00

**Table 17:** Key modal verbs in MRP (in comparison with BNC-S).

No.	Modal verbs	MRP		BNC-S		Keyness	Significance
		Freq.	%	Freq.	%		
55	Must	237	0.32	616	0.06	532.59	0.00
104	Should	254	0.34	1,029	0.11	299.12	0.00
1,317	Can	186	0.25	3,535	0.37	−27.76	0.00
1,343	Could	46	0.06	1,538	0.16	−43.88	0.00
1,346	Might	4	−	691	0.07	−45.07	0.00
1,363	Would	76	0.10	2,524	0.27	−71.87	0.00

## Discussion

### Differences in macro-linguistic features

With regard to macro-linguistic features, there are some differences between the inverse translation SWM and the direct translations CWM and MRP, and the three translations are in a middle position in relation to normalized spoken English texts as well as original spoken English texts. As is shown above, the English translations of Mao’s speeches differ from both normalized spoken English texts and original spoken English texts in the aspects of vocabulary richness, readability, degree of formality, etc.

Those differences in macro-linguistic features may be attributed to different modes of language production. The translated Mao’s speeches belong to constrained English texts, influenced by both the source language and the target language. For one thing, rendered from Chinese into English, the translated Mao’s speeches are unavoidably affected by Mao’s habitual language use. For another, in order to become the cultural facts and occupy a position in the English world (both direct and inverse translations included), the translated Mao’s speeches have to adapt themselves to the norms of the English language. Thus the translated Mao’s speeches come from the double influence of both the source language and the target language. In contrast, both WCH (normalized spoken texts) and BNC-S (original spoken texts) are non-translated, naturally produced English texts, with no interference of the source language. Therefore, the translated Mao’s speeches differ from both normalized spoken English texts and original spoken English texts in certain macro-linguistic aspects.

### Differences in the use of personal pronouns

As to the use of personal pronouns, the three English translations deviate from both normalized spoken English texts and original spoken English texts. This deviation is most noticeable in the use of the first person singular pronoun *I*, which is significantly underused in all the three translations.

Such a phenomenon may be attributed to the source texts and ultimately to the cultural and linguistic differences between China and the West. First, from the cultural perspective, China and the West lay different emphasis on individual status in their culture. The emphasis on collectivism in China has led to a tendency for Chinese leaders to downplay the individuals in speeches, resulting in a higher frequency of the inclusive *we* in Chinese culture. By contrast, the emphasis on individualism in the West allows Western leaders to highlight the power and role of the individuals in speeches, thus resulting in a higher frequency of the first person singular pronoun *I* in western culture. Second, from the linguistic perspective, Chinese as a type of paratactic language tends to use fewer personal pronouns while English as a type of hypotactic language has a preference for personal pronouns to achieve the cohesion and coherence of a text. In Chinese–English translations, due to the influence of the source texts, the English translations may be featured by the underuse of the first person singular pronoun *I*, thus differentiating themselves from both normalized spoken English texts and original spoken English texts.

### Differences in the use of modal verbs

According to Halliday and Matthiessen (2004: 622−624), modal verbs generally can be classified into three types, on the basis of their “value.” As is shown in Table 18, high-value modal verbs include *must, ought to, need, have to,* as well as *be to*; medium-value modal verbs include *will, would, shall,* and *should*; low-value modal verbs include *may, might, can,* and *could*.

**Table 18:** Quantitative values of modal verbs (Halliday and Matthiessen, 2004: 622–624).

Values	Modal verbs
High	Must, ought to, need, have to, be to
Medium	Will, would, shall, should
Low	May, might, can, could

Based on Halliday and Matthiessen’s classification, we can find that, in comparison with normalized spoken English texts and original spoken English texts, all the three English translations prefer the overuse of the high-value modal verb *must* and the medium-value modal verb *should*; on the other hand, the three translations prefer the underuse of medium-value and low-value modal verbs *shall, could, may,* and *would*.

Those features may be attributed to the linguistic characteristics of the source texts. Mao’s speeches are mainly political texts, which contain many non-dialogical imperative sentences and fulfill the “expressive function” as proposed by Newmark (1988: 39−40). According to Sun (1993: 244−248), major modal verbs in Mao’s writings include 必须 (must), 应该/应当/要 (should), and 不能 (must not/should not). Generally speaking, these Chinese modal verbs can be mainly rendered into the high-value modal verb *must* and medium-value modal verb *should* in English. In the process of translating from Chinese into English, the regular use of equivalence/explicitation strategy can lead to the overuse of the high-value modal verb *must* and medium-value modal verb *should* in English translations.

To sum up, the above findings suggest that the macro- and micro-linguistic differences exist not only between direct and inverse translations, but also among translated normalized spoken texts, normalized spoken English texts, and original spoken English texts. Specifically, in terms of macro-linguistic statistics, the average word length of the English translations is higher than that of normalized spoken English texts and that of original spoken English texts; the standardized type/token ratio and average sentence length of the English translations are higher than those of original spoken English texts, but lower than those of normalized spoken English texts. Meanwhile, in terms of the use of personal pronouns, the English translations of Mao’s speeches prefer the underuse of the first person singular pronoun *I*. Furthermore, as far as modal verbs are concerned, the English translations of Mao’s speeches prefer the overuse of *must* and *should* on the one hand, and the underuse of *shall, could, may,* and *would* on the other hand.

## Concluding remarks

Focused on macro-linguistic features, personal pronouns and modal verbs, this study investigates the spoken texts in three English translations of Mao’s writings, taking WCH (Churchill’s speeches, representative of normalized spoken English texts) and BNC-S (spoken texts in BNC Sampler, representative of original spoken English texts) as the reference corpora, so as to explore the features of language use in the inverse and direct translations of Mao’s writings. The findings suggest that, on the one hand, direct and inverse translations share some differences, and on the other hand, the translated of Mao’s speeches can be differentiated from normalized spoken English texts as well as original spoken English texts. The translated of Mao’s speeches are in a middle position in relation to normalized spoken English texts as well as original spoken English texts. This in-betweenness not only exhibits Mao’s creative and idiosyncratic language use, but also reflects the influence of the language transfer from Chinese into English.

Those discrepancies may be due to many factors, including the purposes that the translating activities serve, the functions that the Chinese source texts fulfill in the translating process, the roles of the translators in the translating activities, the attitudes toward the Chinese source texts held by the translators (Wang and Huang, 2020: 14). For example, the inverse translation SWM, initiated by the Chinese government in the early 1960s, aims to transfer Mao Zedong’s thought and introduce China’s revolutionary experience. As authoritative texts in China, Mao’s writings are not only national political documents but also formal speeches which fulfill expressive and vocative functions. It is precisely because of the authoritativeness of Mao’s writings, the inverse translation SWM tends to be more literal and source text-oriented in nature, resulting in a more adequate translation. By contrast, the direct translations CWM and MRP, initiated by the U.S., prefer to consider Mao’s writings as a source of information or the research materials for historical and political studies. Although the source texts are still regarded as authoritative texts, they are, more importantly, seen as informative texts in the translation process. The source texts’ expressive and vocative functions are downplayed, and their informative function comes to the fore. Thus, the direct translations CWM and MRP tend to be more target text-oriented in nature, resulting in more acceptable translations.

To sum up, this study investigates the features of translated normalized spoken texts at the macro-linguistic and micro-linguistic levels. Findings show that, in terms of macro-linguistic statistics, the use of personal pronouns and the use of modal verbs, the translated normalized spoken texts can be differentiated from non-translated original spoken texts and non-translated normalized spoken texts. It should be noted that more indicators (such as deixis, modal particles, cultural-specific terms) are to be explored so as to efficiently distinguish different types of spoken texts.

## Data availability statement

The original contributions presented in the study are included in the article/Supplementary material, further inquiries can be directed to the corresponding author.

## Author contributions

LH designed the research, analyzed the data, and wrote the manuscript. XS collected the data and revised the manuscript. All authors contributed to the article and approved the submitted version.

## Funding

This research was supported by the National Social Science Foundation of China (No. 21&Z D290 & No. 15BYY035).

## Conflict of interest

The authors declare that the research was conducted in the absence of any commercial or financial relationships that could be construed as a potential conflict of interest.

## Publisher’s note

All claims expressed in this article are solely those of the authors and do not necessarily represent those of their affiliated organizations, or those of the publisher, the editors and the reviewers. Any product that may be evaluated in this article, or claim that may be made by its manufacturer, is not guaranteed or endorsed by the publisher.
