# Supplementary material for: A corpus-based investigation of the English translations of Mao Zedong’s speeches
Source: Front Psychol. 2022 Nov 24;13:1071064. doi: 10.3389/fpsyg.2022.1071064 (PMC9731272; doi:10.3389/fpsyg.2022.1071064)
Supplement: Supplementary file 1 [file Data_Sheet_1.pdf]

Appendix 1 | Translated normalized spoken texts in SWM

No.	Title
01	Pay Attention to Economic Work
02	Our Economic Policy
03	Be Concerned with the Well-being of the Masses, Pay Attention to Methods of Work
04	On Tactics Against Japanese Imperialism
05	Win the Masses in Their Millions for the Anti-Japanese National United Front
06	The Role of the Chinese Communist Party in the National War
07	The Question of Independence and Initiative within the United Front
08	Problems of War and Strategy
09	The Orientation of the Youth Movement
10	Reactionaries Must Be Punished
11	Unite All Anti-Japanese Forces and Combat the Anti-communist Diehards
12	New-Democratic Constitutional Government
13	Reform Our Study
14	Speech at the Assembly of Representatives of the Shensi-Kansu-Ningsia Border Region
15	Rectify the Party’s Style of Work
16	Oppose Stereotyped Party Writing
17	Economic and Financial Problems in the Anti-Japanese War
18	Get Organized
19	Serve the People
20	The United Front in Cultural Work
21	We Must Learn to Do Economic Work
22	China's Two Possible Destinies in Chinese Society
23	The Foolish Old Man Who Removed the Mountains
24	The Situation and Our Policy After the Victory in the War of Resistance Against Japan
25	On the Chungking Negotiations
26	The Present Situation and Our Tasks
27	Speech at a Conference of Cadres in the Shansi-Suiyuan Liberated Area
28	Report to the Second Plenary Session of the Seventh Central Committee of the Communist Party of China
29	Methods of Work of Party Committees
30	Address to the Preparatory Meeting of the New Political Consultative Conference

Appendix 2 | Translated normalized spoken texts in CWM

No.	Title
01	Speech at Memorial Service for Mr Li Ting-ming
02	Chairman Mao's Opening Speech at the First Session of the Chinese People's Political Consultative Conference
03	Report at the Land Investigation Movement Rally of Responsible Persons of Soviets of the Ch'u Level and Above of the Eight Counties
04	Smashing the Fifth 'encirclement and Suppression' and Fulfill Soviet Economic Construction Tasks
05	This Year's Election
06	Report of the Central Executive Committee and the People's Committee of the Chinese Soviet Republic to the Second All-soviet Congress

07	Conclusions of Report of Central Executive Committee
08	Closing Speech
09	Speech at Rally in Commemoration of the 13th Anniversary of the Death of Dr. Sun Yat-sen and of Fallen Officers and Men on Battle Front
10	On the New Stage
11	Political Orientation for National General Spiritual Mobilization
12	Speech at Rally of Schools and Youth Bodies in Yenan in Commemoration of the Fourth Anniversary of the '9 December' Movement
13	Comrade Mao Tse-tung Delivers Detailed Report on the Dissolution of the Comintern
14	Comrade Mao Tse-tung Addresses Eastern Anti-fascist Meeting, Calling on All Nations to Strengthen Unity
15	Comrade Mao Tse-tung Delivers a Radio Speech
16	Comrade Mao Tse-tung Sums Up at 1 July Cadres Soiree the Heroic Struggle of 22 Years
17	On Cooperatives
18	Speech at a Cadres Soiree in Yenan in Celebration of the October Revolution Anniversary
19	Comrade Mao Tse-tung Appeals for the Development of Industry to Defeat the Japanese Bandits
20	Chairman Mao Urges Commanders and Fighters to Persist in Advancing the Interests of the People
21	United Front Policy on the Cultural and Educational Fronts
22	Tasks for 1945
23	Speech at Memorial Meeting for China's Revolutionary Martyrs
24	Chairman Mao's Speech Upon Arrival in Chungking
25	Speech at a Political Council Tea
26	Speech at a Reception in the Military Affairs Commission Auditorium

Appendix 3 | Translated normalized spoken texts in MRP

**No.**	**Title**
01	Speech at the Closing Ceremony of the Guangdong Provincial Congress of the Chinese Guomindang
02	Speech at the Welcome Meeting Held by the Provincial Peasants' and Workers' Congresses
03	Speech at the Mass Meeting Convened by the Central Peasant Movement Training Institute in Memory of the Martyrs from Yangxin and Ganzhou
04	Opening Speech at the Conference of Responsible Officials from Eight Xian Regarding the Land Investigation Movement
05	Report at the Land Investigation Movement Meeting Attended by Responsible Soviet Officials at and Above the District Level from Eight Xian
06	Report on Emergency Mobilization
07	Speech at the “August 1” Rally of the Campaign to Mobilize for the War of Resistance
08	Speech at the Evening Send-off Party for the Northwest Battlefield Service Corps
09	Speech at the Yan'an Mass Rally Against Aggression
10	Speech at the Rally in Memory of the Thirteenth Anniversary of Sun Yatsen's Death, and in Honor of the Officers and Men Who Have Died in the War of Resistance
11	Speech at the Opening Ceremony for the Second Session of the Northern Shaanxi Public School
12	Speech at the Opening Ceremony of the First Representative Assembly of the Border Region National Defense Education Commission

13	Speech at the Lu Xun Academy of Arts
14	Talk at the Evening Meeting Commemorating Marx and Sun Yatsen
15	The Political Direction for the General Mobilization of the National Spirit
16	Speech at the Educational Mobilization Meeting for Cadres at Their Posts in Yan'an
17	Speech Given at the Meeting to Commemorate the Third Anniversary of the Anti-Japanese Military and Political University
18	Talk at the Award-Giving Ceremony for Model Youth
19	Speech at the First Representative Conference of the Students National Salvation Association of the Shaanxi-Gansu-Ningxia Border Region
20	Speech at an Evening Welcome Party for the Northern Route Comfort Corps of the National Comfort Corps of China
21	Speech at the Evening Welcome Party for the Army Commander He Zhuguo, Mr. Snow', and Comrades Blestov and Magov
22	Speech at a Meeting of All Circles in Yan'an to Commemorate Stalin's Sixtieth Birthday
23	Speech at the Evening Welcome Party for General Ma Zhanshan, the National Hero
24	Congratulatory Speech at the Mass Meeting Sponsored by the Central Committee of the Chinese Communist Party to Celebrate the Sixtieth Birthday of Comrade Wu Yuzhang
25	Speech at the Opening Ceremony of the Second Agricultural and Industrial Exhibition of the Border Region
26	Speech at the Founding Meeting of the Natural Science Research Association of the Shaanxi-Gansu-Ningxia Border Region
27	Speech at the Founding Meeting of the Yan'an Young People's Association for Promoting Constitutional Government
28	Talk at the First Annual Session of the Yan'an New Philosophy Society
29	Speech at the Meeting Called by the Central Bureau of the Border Region Regarding Economic Self-Sufficiency
30	At the Eastern Anti-Fascist Congress, Comrade Mao Zedong Calls Upon All the Nationalities to Strengthen Their Unity
31	Comrade Mao Zedong Delivers a Broadcast Speech
32	Speech at the Forum on the Reform of Liberation Daily
33	Report on the Question of the Dissolution of the Communist International
34	Comrade Mao Zedong Summarizes Twenty-Two Years of Heroic Struggle at the July 1 Evening Party for Cadres
35	Talk During the Inspection at Nanniwan
36	Talk at the Opening Ceremony for the Second Class of the Central Party School
37	On Cooperatives
38	Speech at the Yan'an Party for Cadres in Celebration of the October Revolution
39	Speech to the Representatives of the Yan'an Masses
40	Comrade Mao Zedong Calls for Developing Industry and Overthrowing the Japanese Bandits
41	Talk at the Opening Ceremony of Yan'an University
42	Chairman Mao Strongly Encourages Fighters to Persist in Serving the People
43	Oral Political Report at the Seventh National Congress of the Chinese Communist Party
44	Speech at the Memorial Assembly for Chinese Revolutionary Martyrs
